# Do Drinking Norms, Motives, and Drinking Behaviors Differ by Age Group among Korean Women?

**DOI:** 10.3390/ijerph19063345

**Published:** 2022-03-11

**Authors:** Aeree Sohn, Sarang Jang

**Affiliations:** Department of Public Health, Sahmyook University, Seoul 01795, Korea; aeree@syu.ac.kr

**Keywords:** drinking, norms, motives, Korean women, age group

## Abstract

Background: Drinking norms and motives accumulate with drinking experience; thus, it is likely that related drinking behaviors will differ with age. This study aimed to predict drinking behaviors by age based on drinking norms and motives in a sample of Korean women. Methods: This exploratory study used a nationwide demographically stratified sample including 1057 women aged 19–59 years. Self-report questionnaires assessed participants’ general drinking frequency and quantity, two drinking norms, and five dimensional motives. The data were analyzed using Spss 26. Results: Descriptive and injunctive norms were the predictors that accounted for the greatest variance in drinking frequency, quantity, binge drinking, and high-risk drinking across all age groups (*p* < 0.001). Descriptive norms predicted all drinking behavior better than injunctive norms and all five motives for all age groups. The effects of each of the five motives differed with age. The enhancement motive was the strongest predictor of the motives for drinking frequency, binge drinking, and high-risk drinking across all age groups. Social and conformity motives predicted only binge drinking. Conclusions: These results suggest that descriptive norms, injunctive norms, and enhancement motives predict drinking behaviors across all age groups, although the relative predictive strength of those variables differed by age.

## 1. Introduction

According to a recent Korea National Health and Nutrition Examination Survey (KNHNES) by the Ministry of Health and Welfare, the amount of alcohol consumed annually by Koreans is decreasing, but the rates of risky and binge drinking among young adults, especially women, is increasing [1]. In 2020, among Korean adults aged 19 years and older, 58.9% (70.2% of men and 47.8% of women) reported consuming alcohol monthly. Further, 51.9% of men and 24.7% of women reported monthly binge drinking (i.e., seven or more drinks for men and five or more drinks for women on one occasion) in the past month [1]. While men’s monthly binge drinking rates have gradually decreased, they have rapidly increased for women, especially those in their 20s and 30s [1]. Further, the gender gap in the rates of binge drinking has gradually decreased from 33.2% (53.7% for men vs. 20.5% for women) in 2007 to 27.2% (51.9% for men vs. 24.7% for women) in 2020. Binge drinking and heavy drinking are associated with social and economic burdens and are risk factors for chronic diseases, such as cardiovascular disease, liver disease, obesity, and metabolic syndromes [2]. While fewer women overall drink alcohol, those who drink heavily are more likely to have problematic drinking behaviors than men. Previous research has primarily focused on physiological vulnerabilities and social responsibilities when studying women’s drinking behaviors, but traditional gender roles have changed as the number of women in labor and economic markets and obtaining higher education has increased [3]. Further, regardless of gender, young adults have more risk factors for problematic drinking than middle-aged and older adults. In addition, marriage status, income, and employment are known to affect women’s alcohol use [4].

Drinking, especially binge drinking or high-risk drinking, is largely influenced by sociocultural factors, including social drinking norms and motives, traditional drinking patterns, and a country’s alcohol policies [5]. Norms are subjective perceptions unless they have been codified into laws, such that normative beliefs are what one “thinks” another person wants to be done. There are two types of norms—descriptive and injunctive. Descriptive norms are the perceptions of the extent to which a particular behavior occurs in society [5,6,7], such as the perceived prevalence of drinking behaviors occurring around them [5,8]. Drinking norms and motives are related to individual characteristics, such as gender and age, that influence choices about alcohol consumption and drinking behaviors [5,9]. The relationship between drinking norms and motives may differ according to age by gender. The role of age is more than simply a biological aging process affecting health outcomes but also a reflection of the accumulated sociocultural experiences of an individual over the course of their life. There might be an interaction between age and gender, such that gender moderated the relationship between age and drinking norms and motives [10,11].

According to Riley’s age stratification theory [10], each age cohort shares social roles and experiences. Age is cross-sectional, as people may experience the same historical events or culture in their childhood or adulthood at critical points in time. The social consequences of age might affect attitudes and behaviors differently across each age group [10].

Korea has one of the most rapidly changing societies worldwide, and the gender roles and social responsibilities of women have changed dramatically in the past forty years. While social expectations and life experiences differ for each age cohort, analyses of drinking patterns among women by age have not received sufficient attention in Korea [12].

Research has contended that the main reason for drinking alcohol in Korea is to reduce work-related stress, as rapid economic growth leads Koreans to work long hours [3,8]. Company-wide drinking, referred to as *hoesik*, meaning “staff dining” or “company dining”, is very common in Korea. Ko and Sohn [8] described it as “eating/drinking fests involving multiple rounds at multiple venues.” Some articles [5,8] have stated that drinking culture and motives have changed from “company dining” to “social drinking due to personal pressures.” In particular, one reason for drinking among younger generations and women is to enhance their activities or increase their fun rather than relieve stress. In many studies, the drinking motives discriminate between different drinking behaviors among men and women [13]. Enhancement and coping motives have been significantly related to binge drinking, while social motives have been linked to moderate drinking [13]. However, this kind of study to develop prevention strategies is currently neglected in public health interventions among Korean women.

Many studies of women’s alcohol use in Korea have focused on differences between men and women; therefore, there has been less research on other individual factors, such as social norms and motives at different ages [4,8,14]. Since many studies on drinking motives tend to focus on college-aged students or adolescents [15], more evidence is necessary to understand how drinking motives relate to drinking behavior, such as drinking frequency and quantity, across different age groups, especially middle-aged and older-aged adults. Some studies have shown that the drinking motives of the younger-aged elderly are different from those of the older-aged elderly [16,17]. In recent years, young women have begun to drink at an earlier age and consume more alcohol than women in previous generations. Women’s alcohol-use-related problems have risen with the increase in the number of young women drinkers [18,19]. Women who have problems with alcohol are reluctant to seek help for their alcohol use owing to severe discrimination and stigma toward heavy drinking among women in South Korea [4,14]. Therefore, this study aimed to examine women’s drinking patterns in relation to age, norms, motives, and drinking behaviors. Based on the previous empirical research, two hypotheses were developed. The first hypothesis was that descriptive and injunctive norms would predict drinking frequency, quantity, binge drinking, and high-risk drinking and they would differ by age. The second was that drinking motives would predict drinking frequency, quantity, binge drinking, and high-risk drinking and they would differ by age.

## 2. Materials and Methods

### 2.1. Participants and Procedures

Demographically proportioned stratified sampling was conducted using a panel of participants enrolled in June 2021 by the domestic company “M” to recruit the study participants. Respondents were screened for eligibility using questions regarding their age and region. The allocated female sample size of 1100 was stratified by age and region to obtain a nationally representative sample. A cleaning process was performed for the derived data and subsequently, 1067 cases were analyzed. Their mean age was 40.5 years (*SD* = 11.2). To compare age groups for the purposes of the analysis, the respondents were divided into four age groups—those 20–29 (*n* = 239, 22.4%), 30–39 (*n* = 225, 21.1%), 40–49 (*n* = 290, 27.2%), and 50–59 (*n* = 313, 29.3%). Fifty-one percent of participants were married or living with a partner, 22.8% were high school graduates or less, 12.0% had a low monthly income (up to KRW 1999), 68.9% had a middle income (KRW 2000–6999), and 19.1% had a high income (KRW 7000 or more).

Prior to conducting the survey with a prepared questionnaire, the study protocol was approved by our institutional review board (2-1040781-A-N-012021023HR). Informed consent was obtained from all participants. The survey was conducted from June to July 2021 using an online survey. 

### 2.2. Measures

#### 2.2.1. Outcome Variables: Drinking Behaviors

*Drinking frequency and quantity*. In this study, the data available before the onset of the COVID-19 pandemic were used. Participants were asked how often they had consumed alcohol over the past year (i.e., 2019) and how much they usually drank on each occasion. For the regression analyses, these variables were converted to the number of standard drinks. 

*Binge drinking and high-risk drinking.* Binge drinking is considered risky because it can increase blood alcohol concentration dramatically within a short period [2]. The definition of binge drinking in South Korea is regarded as five or more standard drinks for women (seven or more for men) per drinking occasion over the past month [1]. One standard drink in Korea contains 7–8 g of ethanol [20]. High-risk drinking for women in South Korea is defined as two or more binge drinking occasions per week with more than five standard drinks per drinking occasion [20]. 

#### 2.2.2. Independent Variables: Norms and Motives

Descriptive norms refer to the perceptions of the drinking frequency and quantity of others. Injunctive norms refer to the perceived approval of drinking and represent perceived moral rules [18,19]. Injunctive norms and drinking motives could differ by a county’s drinking culture [5]. Sohn developed a measure to assess the injunctive norms and drinking motives for Koreans, which was modified for this study [21]. The items for injunctive norms and drinking motives were identical to those used in Sohn’s study [21], which classified 12 items related to drinking motives along three dimensions reflecting individual (enhancement and coping motive, internal motive), social (social and conformity motive, external motive), and environmental motives (external motive). As there were high intercorrelations between enhancement and coping (internal factor) and social and conformity (external factor), Sohn combined these scales into two factors [21]. Three additional items were developed according to five conceptually and empirically distinct reason dimensions (i.e., enhancement, coping, social, conformity, and environmental). Cooper [22] developed the Drinking Motive Questionnaire with 4 drinking motive categories—enhancement (internal, positive), coping (internal, negative), social (external, positive), and conformity (external, negative). Considering Cooper’s 4 categories and Sohn’s environmental category, the Drinking Motive Questionnaire for Koreans was developed and included 15 items on five dimensions with three items each. The scale was tested in a previous study [23], which showed its suitability for modeling.

*Descriptive norms and injunctive norms.* Descriptive norms (i.e., perceptions concerning friends’ behavior) were measured by assessing how frequently respondents thought their friends drank alcohol. Injunctive norms included positive perceptions in nine different situations (e.g., whether respondents considered it acceptable to drink at a park, on a mountain, during the day, when under-aged, or alone) [21]. In this study, descriptive norms were measured by assessing how often respondents’ thought their friends and colleagues drank alcohol. The items were rated on a five-point Likert-type scale, the injunctive norms scores were summed to yield the total score; the higher the score, the more acceptable certain drinking behaviors were considered. The Cronbach’s α was 0.78. The instructions included a statement that respondents who did not drink at all should answer these questions to include the responses of abstainers in the data.

*Drinking motives*. Drinking motives were measured using 15 self-reported items on five subscales with three items each. The 5 subscales were enhancement (e.g., drinking to feel happy), coping (e.g., drinking to overcome stress), social (e.g., drinking to become friends quickly), conformity (e.g., drinking for work-related gatherings), and environmental context (e.g., drinking because there are many places to drink everywhere) motives for drinking. The participants were asked to rate how often each statement reflected their reasons for drinking over the last 12 months on a five-point Likert-type scale ranging from 1 (*strongly disagree*) to 5 (*strongly agree*).

Except for lifetime abstainers who had never consumed alcoholic beverages, 991 respondents answered the drinking motive items to obtain the exact drinking motives. Higher scores indicated greater agreement with the enhancement, coping, social, conformity, and environmental drinking motives. Cronbach’s coefficients for the enhancement, coping, social, conformity, and environmental (accessible) drinking motives scales were 0.70, 0.73, 0.86, 0.79, and 0.82, respectively.

#### 2.2.3. Sociodemographic Variables

Sociodemographic variables were categorized in detail; however, they were analyzed as dichotomous variables for further analyses. Sociodemographic variables consisted of the age group (20–39, vs. 40–59), marital status (never married, separated, divorced, and widowed, vs. married or living with a partner), education (<2-year college degree vs. ≥2-year college degree), job status (employed, temporarily employed, self-employed, vs. unemployed), monthly household income (low—up to 1999 k KRW vs. middle—2000 k–6999 k KRW and high—7000 k KRW or more).

### 2.3. Data Analysis

SPSS statistics software package version 26.0 (IBM Corp., Armonk, NY, USA) was used for the statistical analyses. The characteristics of the different age groups were analyzed using descriptive statistics (frequency, percentage, means, and standard deviation). Drinking behaviors, norms, and motives variables were analyzed using ANOVAs and chi-square tests by age group.

Multiple and logistic regression analyses assessed differences and influences of demographic variables, norms, and motives on the frequency of drinking occasions and quantity of drinks consumed by age group. The numbers of participants who reported binge and high-risk drinking were insufficient to conduct analyses by age group. Before the multiple and logistic regression, significant variables were identified using correlation analysis. 

## 3. Results

### 3.1. Drinking Behaviors

Table 1 presents the drinking behaviors across the four age groups (i.e., 20s, 30s, 40s, and 50s). The most frequent pattern among all age groups was drinking two to four times per month (27.3%). Monthly drinking was reported in 56.4% (12.9% + 27.3% + 16.2%) of respondents. About 22% drank less than once per month, and one-fifth (21.2%) were abstainers. The quantity of alcohol consumed on each occasion was assessed by the number of standard drinks. The mean drinking quantity across all age groups was 4.06 (*SD* = 4.54) standard drinks. 

Drinking behaviors were significantly different in the frequency and quantity of drinking occasions with age. Abstention rates differed by age group and were 19.2%, 16.0%, 18.6%, and 28.8% for those in their 20s, 30s, 40s, and 50s, respectively. The prevalence of drinking was the lowest among adults in their 50s. The mean drinking quantity was significantly higher in younger versus older age groups (*p* < 0.001).

The proportion of participants reporting binge drinking significantly decreased by age (*p* < 0.001)—19.4% of those in their 50s reported binge drinking compared to 51.3% of those in their 20s. High-risk drinking was significantly more frequent in younger age groups than older (*p* < 0.001), with the percentage of high-risk drinkers peaking in their 20s (15.9%), followed by those in their 30s (12.4%).

### 3.2. Descriptive and Injunctive Drinking Norms

Table 2 shows that the descriptive and injunctive norms regarding drinking differed by age group. When participants were asked how often they thought their friends and colleagues drank alcohol, the highest response was that they drank two to four times per month (33.6%), followed by two or more times per week (25.7%).

As shown in Table 2, respondents reported that their friends and colleagues drank more frequently than they did (*p* < 0.001). Friends’ drinking frequency of two to four times per month differed by age group, being the highest for participants in their 20s (38.9%), followed by those in their 30s (34.2%), those in their 50s (33.5%), and those in their 40s (28.6%).

Among the drinking behaviors considered acceptable by those in their 20s vs. those in their 50s, the highest reported percentage was for “drinking alone” (79.1% vs. 40.6%), followed by “getting drunk” (59.4% vs. 15.3%), “drinking during the day” (58.6% vs. 8.3%), and “drinking in a convenience store” (31.4% vs. 13.4%). For all statements, except “Crimes committed while drunk can be forgiven” and “It is discourteous to refuse a drink offered by someone else”, respondents in their 20s and 30s were more likely to agree significantly than those in their 40s and 50s (*p* < 0.001).

As seen in Table 2, respondents in their 20s and 30s (means of injunctive norms 2.45, 2.35, respectively) showed higher agreement than those in their 40s and 50s (means of injunctive norms 2.14, 1.99, respectively; *p* < 0.001). 

### 3.3. Drinking Motives

The drinking motives of each age group are presented in Table 3. Among female drinkers in their 20s, the highest number of participants agreed with “to become friends quickly and to get along with them” (61.8%), followed by “it lifts the mood of the meeting” (60.8%), “to feel good and happy” (58.5%), and “to overcome work and life stress” (54.7%). Among those in their 30s, the highest number of participants agreed with “to feel good and happy” (60.9%), followed by “to overcome work and life stress” (58.0%), “to become friends quickly and to get along with them” (48.3%), and “it lifts the mood of the meeting” (47.8%). Among those in their 40s, the highest number of participants agreed with “to feel good and happy” (58.1%), followed by “it lifts the mood of the meeting” (55.9%), “to become friends quickly and to get along with them” (50.5%), and “to overcome work and life stress” (49.8%). Among those in their 50s, the highest number of participants agreed with “it lifts the mood of the meeting” (57.3%), followed by “to feel good and happy” (52.2%), “to become friends quickly and to get along with them” (51.2%), and “to overcome work and life stress” (48.1%). The means of the five dimensional motives for all age groups were in the same order.

The social motive (*M =* 3.18) ranked the highest among the five motives, followed by enhancement (*M =* 2.85), coping (*M =* 2.54), conformity (*M =* 2.18), and environmental (*M =* 1.92). The five motives ranked in the order of social > enhancement > coping > conformity > environmental in all age groups. Different levels were shown by age group, and the highest level of social motive was found among those in their 20s. The enhancement motive had the highest score among those in their 30s, the coping motive among those in their 40s, and the conformity and environmental motives among those in their 50s.

### 3.4. Multiple Regression Analyses Predicting Drinking Frequency and Quantity across Age Groups

A multiple regression analysis was conducted to determine the factors associated with the frequency of drinking and quantity of alcohol across age groups (Table 4). The sociodemographic variables (education status, marital status, household income), smoking status, two norms, and five dimensional motives were entered into the model. The full regression model accounted for 55%, 61%, 57%, and 34% of the variance by age group (20s, 30s, 40s, and 50s, respectively) in drinking frequency (*R*^2^ = 0.55, 0.61, 0.57, 0.34, respectively, *p* < 0.001). The quantity of alcohol consumption accounted for 36%, 37%, 31%, and 21% of the variance by age group—20s, 30s, 40s, and 50s (*R*^2^ = 0.36, 0.37, 0.31, 0.21, respectively, *p* < 0.001).

The regression analyses showed that descriptive norms (i.e., those who thought their friends drank frequently) were the most important factors and positively related to the drinking frequency and quantity for all age groups. Injunctive norms were significantly and positively related to drinking frequency and quantity for most age groups.

Regarding the five motives, the enhancement motive was the most important motive and positively related to drinking frequency and quantity for most age groups. The coping motive was not significantly correlated with the frequency and quantity of drinking among all age groups. The social motive was significantly and positively associated with drinking quantity for women in their 20s. The conformity motive was significantly negatively correlated with drinking frequency and quantity in most age groups. Overall, these findings suggest that those with a high level of conformity were more likely to drink less in terms of frequency and amount. The environmental motive was significantly positively correlated with drinking frequency for women in their 40s. 

Of the sociodemographic variables, the regression analysis revealed that only household income had a significant positive association with drinking frequency only for those in their 30s. Additionally, smoking was significantly associated with the quantity of drinking for those in their 20s, 30s, and 40s.

### 3.5. Logistic Regression Analyses Predicting Binge Drinking and High-Risk Drinking

As shown in Table 5, in the full sample, the full logistic regression model accounted for 35% and 37% of the variance in binge drinking and high-risk drinking, respectively (Nagelkerke *R*^2^ = 0.35, 37). Binge drinking rates were significantly associated with age (*p* < 0.001), education (*p* < 0.001), smoking status (*p* < 0.001), descriptive norms (*p* < 0.001), injunctive norms (*p* < 0.001), enhancement motive (*p* < 0.001), social motive (*p* < 0.05), and conformity motive (*p* < 0.05). Those who were younger, high school graduates, smokers, and had a high level of descriptive norms (those who thought their friends drank frequently) and injunctive norms (those who are socially acceptable to specific drinking behaviors), enhancement motives, social motives, and low levels of conformity were more likely to be binge drinkers.

In the full sample, the probabilities showed a significant association between a high-risk drinking rate and marital status (*p* < 0.05), descriptive norms (*p* < 0.001), injunctive norms (*p* < 0.001), and the enhancement motive (*p* < 0.001). High-risk drinkers were more likely to be divorced or separated, have high levels of descriptive norms, injunctive norms, and the enhancement motive. Descriptive norms, injunctive norms, and the enhancement motive were significant for both binge drinking and high-risk drinking. Age, education, household income, smoking status, and social, conformity, coping, and environmental motives were not significantly related to high-risk drinking; however, some of those variables (age, education status, smoking, social and conformity) approached a significant level for binge drinking.

## 4. Discussion

### 4.1. Dinking Behaviors

This study examined drinking behaviors (i.e., frequency, quantity, binge drinking, and high-risk drinking), two norms (i.e., descriptive and injunctive norms), and five drinking motives by age group. Factors affecting the frequency, quantity, binge drinking, and high-risk drinking were identified. Drinking frequency and quantity significantly differed by age, with the highest rates among those in their 30s, 20s, 40s, and 50s, in that order. Binge drinking and high-risk drinking peaked among those in their 20s, with much larger differences in the rate of binge drinking among those in their 20s and 50s.

Our findings are similar to those of the KNHNES, which found that binge or high-risk drinking was more frequent in younger age groups compared to older age groups, while monthly drinking, binge drinking, and high-risk drinking in men did not significantly differ by age [1]. In the KNHNES sample, younger women started drinking earlier than older women [1]. One likely reason for this difference is related to the economic development of Korea, which has led to a rapid increase in women’s drinking, possibly because of women’s educational and employment gains. The monthly and high-risk drinking rates from the KHNES showed a decreasing trend between 2007 and 2019 in men after a peak in 2011, while women showed an increasing trend, reaching a peak in 2017 [1]. In a panel analysis by Kim et al. [4] using data from the Korea Labor Panel, there were significant differences in drinking behaviors and amounts between men and women. Several studies have reported that the generational differences in alcohol use were more prominent among women than men in Korea [4,8,18]. Thus, additional attention should be given to the increases in women’s alcohol use in Korea.

### 4.2. Drinking Norms

This study found that descriptive and injunctive norms regarding drinking differed by age group. Descriptive norms were the strongest predictor across all age groups, especially younger age groups. The respondents believed that their friends and colleagues drank more frequently than they did. Respondents who believed that their friends drank frequently were more likely to drink frequently themselves. They also drank more and were significantly more likely to be binge drinkers or high-risk drinkers. Among the respondents, norms served as a standard for judging the appropriateness of their actions by observing others’ drinking behavior [5,8,13]. Consistent with prior research, the individuals in our study who overestimated the frequency and quantity of alcohol use by others were the heaviest drinkers [13]. In more practical terms, the results of this analysis have implications for the design of changing norms of effective alcohol-reduction policies.

Injunctive norms are perceptions regarding which specific drinking behaviors are socially acceptable [19]. The regression analysis revealed that injunctive norms were the second most important factor associated with the frequency and quantity of alcohol use for all age groups. Respondents who reported high acceptable drinking perceptions in society were more likely to be binge drinkers or high-risk drinkers for all age groups. Younger women in their 20s and 30s had higher perceptions that certain drinking behaviors were acceptable in society than those in their 40s and 50s. For example, of the drinking behaviors considered acceptable by those in their 20s vs. those in their 50s, the highest-rated behaviors were “drinking alone” (79.1% vs. 40.6%) and “drunkenness” (59.4% vs. 15.3%). These differences in norms led to different drinking behaviors. 

Drinking alone was perceived more favorably by younger women than older women in this study. Recently, the popularity of solitary drinking has grown in Korea [20,21] due to the increasing number of people living alone and the implementation of social distancing measures because of COVID-19. Prior research has shown that solitary drinking is associated with greater alcohol consumption and more alcohol-related problems than drinking with others [24,25]. Solitary drinking is not in itself a problematic behavior, but it should be given further attention [24].

### 4.3. Drinking Motives

In this study, there were large differences in drinking motives between the younger and older respondents, although the ranking of the motives was the same. The five dimensional motives ranked in the order of social > enhancement > coping > conformity > environmental for all age groups. However, in terms of the type of drinking motive, the age group rating for the highest-level motive differed, with those in their 20s having the highest social motive rating, those in their 30s having the highest enhancement motive rating, those in their 40s having the highest coping motive rating, and those in their 50s having the highest ratings for the conformity and environmental motives. Younger women had a higher motivation for drinking to enhance positive emotions and cope with negative ones compared to older women, while older women had a higher motivation to drink due to social pressure and environmental stimuli or temptation. These findings suggest that the drinking culture of enjoyment represents a leisure activity for younger adults in Korea. In terms of drinking frequency and quantity, those with a high level of enhancement and low level of conformity were likely to drink more. Although the social motive was rated the highest, it did not predict drinking frequency and quantity. Since all Korean women, regardless of age, had high social motive levels, there was less variation, which did not predict their drinking behaviors.

In particular, the enhancement motive of the five motives significantly predicted both binge and high-risk drinking. These findings are consistent with research reporting that enhancement motives are the frequent drinking motivation reported among adolescents [13], with young adults [15,26] engaging in binge drinking. The social and conformity motives predicted only binge drinking and not high-risk drinking. The results showing that the coping and environmental motives did not predict drinking behavior were inconsistent with findings from other countries [13,27]. A study conducted on Americans showed that conformity and social motives had different relationships by age group [15]. In the study, the conformity motives predicted greater hazardous drinking for younger-aged adults, while the conformity and social motives predicted less hazardous drinking for middle-aged adults [15]. Differences in drinking motives have been reported to be distinct for countries or generations, suggesting that one’s drinking culture may affect an individual’s behaviors [13,15,27]. In this context, the motivational aspects of drinking behaviors may represent an appropriate target for intervention to reduce binge drinking and high-risk drinking among women of different ages.

In addition, respondents who were divorced or separated were more likely to engage in high-risk drinking; therefore, special attention should be paid to these populations. Many studies regarding health behaviors such as smoking, alcohol consumption, and physical activities have found large gaps across age, income, education, and occupation groups [4,28]. Distress from being in a vulnerable group and being isolated in society may lead to high-risk drinking or smoking, which are both readily available [4,14,28]. Previous studies have reported that economic pressure weakens individuals’ coping mechanisms, increasing their vulnerability to stressful life events, and psychological vulnerabilities are positively related to drinking motivation and consequently affect problematic drinking behavior [4].

### 4.4. Limitations

This study had several limitations. The data reflected the drinking frequency and quantity that were assessed before the onset of the COVID-19 pandemic, so they were likely to have a recall bias. The COVID-19 pandemic has led to considerable changes in drinking patterns worldwide, including in South Korea. Drinking frequency and quantity in most groups has decreased owing to social distancing measures in South Korea [29].

The drinking motives scale used in this study had some limitations related to its psychometric validation. In Sohn’s study [21], there were high intercorrelations between the five motives, with the enhancement motive being highly related to the coping motive and the conformity motive being related to the environmental motive. Thus, Koreans who drink alcohol to enhance positive emotions also reported drinking to cope with negative ones. Further, Koreans who drink due to social pressure reported drinking due to environmental drinking stimulation. Thus, the Korean version of the drinking motive questionnaire should be developed according to five conceptually and empirically distinct reason dimensions. 

## 5. Conclusions

The study findings indicate that young women tend to consume more alcohol and drink more frequently than older women. The increased alcohol consumption by young women has led to a greater proportion of binge drinking and high-risk drinking among young women compared to older women. Since women who engage in high-risk drinking are more vulnerable to alcohol dependence or alcoholism than men [4,18], early diagnosis and early intervention strategies to prevent alcohol problems should be developed. The concentration of stores and bars in South Korea, therefore, represents a potential public health challenge that could be addressed through alcohol policy reform.

The study findings indicated that the descriptive and injunctive norms played an important role in influencing the frequency and quantity of drinking for all age groups. Respondents who reported high levels of injunctive norms (e.g., high perceptions of drunkenness being acceptable, adolescents’ drinking, drinking in public places) reported more drinking behaviors. These norms could be changed by public campaigns to reduce risky drinking behaviors. A government-led campaign is needed to address descriptive norms and decrease excessive drinking. In addition, people should be informed of the criteria for low-risk drinking.

## Figures and Tables

**Table 1:** Drinking behaviors across age groups (*n* = 1067).

	Age Group					
	Total	20s	30s	40s	50s	χ^2^ or *F* (*p*)
	(*n* = 1067)	(*n* = 239)	(*n* = 225)	(*n* = 290)	(*n* = 313)	
	%	%	%	%	%	
Drinking Frequency						χ^2^ = 42.28 (0.001)
Drink nothing at all	21.2	19.2	16.0	18.6	28.8	
Less than once per month	22.4	22.2	18.7	22.8	24.9	
Once per month	12.9	12.6	12.0	16.6	10.5	
Two to four times per month	27.3	27.2	31.1	25.5	26.2	
Two or more times per week	16.2	18.8	22.2	16.6	9.6	
Drinking quantity on each occasion (# of standard drinks)						
*M* (*SD*)	4.06 (4.54)	5.81 (5.46)	4.87 (5.12)	3.60 (4.02)	2.56 (2.95)	*F =* 28.38 (<0.001)
Monthly drinker	56.4	58.6	65.3	58.6	46.3	χ^2^ = 21.26 (<0.001)
Monthly binge drinker	32.6	51.3	38.1	27.3	19.4	χ^2^ = 68.92 (<0.001)
High-risk drinker	10.0	15.9	12.4	9.3	4.5	χ^2^ = 21.46 (<0.001)

**Table 2:** Descriptive and injunctive norms across age groups (% of agreement, *N* = 1067).

Norms	Age Group					
	Total	20s	30s	40s	50s	χ^2^ or *F* (*p*)
	(*n* = 1067)	(*n* = 239)	(*n* = 225)	(*n* = 290)	(*n* = 313)	
	%	%	%	%	%	
Descriptive norms: Friends’ drinking frequency						χ^2^ =35.65 (<0.001)
Drink nothing at all	14.3	11.3	14.2	13.8	17.3	
Less than once per month	15.0	13.0	12.9	16.2	16.9	
Once per month	11.4	10.5	9.3	14.1	11.2	
Two to four times per month	33.6	38.9	34.2	28.6	33.5	
Two or more times per week	25.7	26.4	29.3	27.2	21.1	
Injunctive norms ^†^						
It is acceptable to drink in a park or on a mountain after hiking.	10.5	20.9	14.7	7.6	2.2	χ^2^ = 77.19 (<0.001)
It is acceptable to drink during the day.	30.6	58.6	45.3	20.0	8.3	χ^2^ = 240.27 (<0.001)
It is acceptable to get drunk.	34.3	59.4	42.7	27.6	15.3	χ^2^ = 147.22 (<0.001)
It is acceptable for high school students to drink.	2.3	3.8	3.1	2.1	1.0	χ^2^ = 17.35 (0.008)
It is acceptable to drink alone.	61.2	79.1	76.4	56.9	40.6	χ^2^ = 122.93 (<0.001)
Crimes committed while drunk can be forgiven.	0.8	1.3	0.4	0.7	1.0	χ^2^ = 5.68 (0.460)
It is discourteous to refuse a drink offered by someone else.	2.4	1.7	4.0	2.1	2.2	χ^2^ =3.36 (0.763)
It is acceptable to drink in a convenience store.	20.0	31.4	22.2	15.9	13.4	χ^2^ = 50.78 (<0.001)
Crimes committed while being intoxicated can receive less severe sentences.	0.6	1.3	0.4	0.0	0.6	χ^2^ = 10.12 (.120)
*M* (*SD*)	2.21 (0.49)	2.45 (0.47)	2.35 (0.46)	2.14 (0.47)	1.99 (0.45)	*F* = 53.73 (<0.001)

Note: ^†^ % of agree and strongly agree (5-point scale from *strongly disagree* to *strongly agree*); *M,* mean; *SD,* standard deviation.

**Table 3:** Five drinking motives across age groups (% of agreement and mean; *n* = 991) ^†^.

	Age Groups					
I Drink....	Total	20s	30s	40s	50s	χ^2^ or *F* (*p*)
	(*n* = 991)	(*n* = 212)	(*n* = 207)	(*n* = 279)	(*n* = 293)	
Five dimensional motives	%	%	%	%	%	
1. Enhancement motives						
to feel good/happy.	57.0	58.5	60.9	58.1	52.2	χ^2^ = 11.46 (0.075)
because there are few things to enjoy except drinking.	19.7	18.4	24.6	21.5	15.4	χ^2^ = 10.71 (0.098)
because alcohol makes food taste better.	30.8	33.5	35.7	30.5	25.6	χ^2^ = 21.78 (0.001)
2. Coping motives						
to overcome work and life stress.	52.1	54.7	58.0	49.8	48.1	χ^2^ = 22.78 (0.001)
because I want to fall asleep quickly when I cannot fall asleep easily.	19.2	13.2	17.9	20.1	23.5	χ^2^ = 18.81 (0.004)
when I want to get rid of my fears and be brave.	14.1	14.6	14.0	12.5	15.4	χ^2^ = 9.32 (0.157)
3. Social motives						
to become friends quickly and to get along with them.	52.7	61.8	48.3	50.5	51.2	χ^2^ = 12.26 (0.056)
to deal with difficult relationships.	32.1	34.4	28.5	29.4	35.5	χ^2^ = 8.21 (.223)
because it lifts the mood of the meeting.	55.7	60.8	47.8	55.9	57.3	χ^2^ = 9.39 (.153)
4. Conformity motives						
because of work-related gatherings.	21.4	15.1	18.4	21.9	27.6	χ^2^ = 19.34 (0.004)
because my friends force me to drink.	8.2	4.2	7.2	6.8	13.0	χ^2^ = 53.28 (<0.001)
to avoid being left out.	17.3	17.0	14.5	17.6	19.1	χ^2^ = 6.98 (0.323)
5. Environmental context motives						
because there are many places to drink everywhere.	13.5	14.2	14.0	11.5	14.7	x^2^ = 5.96 (0.428)
to enjoy low-cost recreation because the price of alcohol is low.	6.7	6.1	4.8	5.7	9.2	χ^2^ = 26.98 (<0.001)
because an alcohol advertisement (TV, poster, etc.) makes me want to drink	5.7	5.2	4.3	6.1	6.5	χ^2^ = 25.05 (<0.001)
Five dimensional motives	Total	20s	30s	40s	50s	
	M	M	M	M	M	
1. Enhancement	2.85	2.78	2.95	2.94	2.75	*F* = 4.65 (0.003)
2. Coping	2.54	2.37	2.57	2.61	2.58	*F* = 3.18 (0.023)
3. Social	3.18	3.22	3.11	3.18	3.19	*F* = 0.50 (0.680)
4. Conformity	2.18	1.97	2.06	2.22	2.40	*F* = 10.66 (<0.001)
5. Environmental (or Accessible)	1.92	1.71	1.82	1.97	2.08	*F* = 9.56 (<0.001)

Note: ^†^ Respondents other than lifetime abstainers answered. % of agree and strongly agree (a 5-point scale from strongly disagree to strongly agree).

**Table 4:** Multiple regression analyses predicting drinking frequency and quantity across age groups.

	Frequency				Quantity			
	20s	30s	40s	50s	20s	30s	40s	50s
	(*n* = 212)	(*n* = 207)	(*n* = 279)	(*n* = 293)	(*n* = 212)	(*n* = 207)	(*n* = 279)	(*n* = 293)
	β	β	β	β	β	β	β	β
Demographic Variables								
Education	0.06	−0.04	0.03	−0.03	−0.06	−0.01	−0.09	−0.12 *
Marital Status	0.05	0.07	0.01	0.01	0.10	−0.10	−0.04	−0.04
Household Income	0.02	0.14 ***	−0.02	−0.01	0.00	0.06	−0.03	−0.03
Smoking	0.02	0.03	0.04	0.02	0.12 *	0.12 *	0.16 **	0.06
Norms								
Descriptive Norms	0.45 ***	0.58 ***	0.47 ***	0.43 ***	0.32 ***	0.31 ***	0.21 ***	0.27 ***
Injunctive Norms	0.21 ***	0.07	0.16 ***	0.17 ***	0.19 **	0.12 *	0.18 **	0.21 ***
Motives								
Enhancement	0.30 ***	0.19 ***	0.29 ***	0.25 ***	0.13	0.29 ***	0.24 **	0.24 ***
Coping	−0.01	0.03	−0.01	0.08	0.04	−0.13	−0.13	−0.08
Social	0.05	0.07	−0.02	0.01	0.15 *	0.11	0.11	0.00
Conformity	−0.10	−0.27 ***	−0.43 ***	−0.21 **	−0.11	−0.14	−0.33 ***	−0.16
Environmental	−0.04	0.04	0.17 **	−0.07	−0.08	0.04	0.16	0.04
*R* ^2^	0.55	0.61	0.57	0.34	0.36	0.37	0.31	0.21
F (*p*)	22.02 (<0.001)	27.82 (<0.001)	31.99 (<0.001)	12.96 (<0.001)	10.01 (<0.001)	10.48 (<0.001)	11.06 (<0.001)	6.96 (<0.001)

Note: Marital status (married = 1, single/divorced/widow = 0), smoking (smoker = 1, non-smoker = 0); $R^{2}$ = 19.8%; * *p* < 0.05, ** *p* < 0.01, *** *p* < 0.001.

**Table 5:** Logistic regression analyses predicting binge drinking and high-risk drinking in total.

	Binge Drinking			High-Risk Drinking		
	OR	Lower 95% CI	Higher 95% CI	OR	Lower 95% CI	Higher 95% CI
Demographic Variables						
Age Group	0.56 ***	0.38	0.82	0.97	0.55	1.72
Education	0.55 ***	0.36	0.83	1.08	0.57	2.05
Marital Status	0.88	0.60	1.31	0.59 *	0.33	1.07
Household Income	1.00	0.58	1.73	2.01	0.79	5.10
Smoking	2.92 ***	1.56	5.47	1.99	0.98	4.03
Norms						
Descriptive norms	1.53 ***	1.34	1.75	2.83 ***	2.15	3.73
Injunctive norms	2.94 ***	1.92	4.49	2.56 ***	1.38	4.74
Motives						
Enhancement	1.90 ***	1.42	2.54	2.08 ***	1.34	3.22
Coping	0.93	0.73	1.18	0.81	0.56	1.15
Social	1.27 *	1.01	1.59	1.24	0.87	1.77
Conformity	0.75 *	0.57	0.99	0.78	0.53	1.15
Environmental	0.88	0.65	1.20	1.06	0.68	1.63
Nagelkerke *R*^2^	0.35			0.37		
−2 log likelihood	841.80			417.72		

Note: Age group (40–59 = 1, 19–39 = 0), education (≥ college graduates = 1, ≤ high school graduates = 0), marital status (married = 1, single/divorced/widow = 0), household income (≥KRW 2000 = 1, ≤ KRW 1999 = 0), smoking (smoker = 1, non-smoker = 0); $R^{2}\;$ = 19.8%; OR = odds ratio; * *p* < 0.05, *** *p* < 0.001.

## Data Availability

Any queries regarding the data used in this study may be directed to the corresponding author. The dataset used in the present study is available on reasonable request.
